# Single dose gadobenate dimeglumine for imaging chronic of myocardial infarction in comparison to double dose gadopentetate dimeglumine

**DOI:** 10.1186/1532-429X-11-S1-P235

**Published:** 2009-01-28

**Authors:** Kerstin U Bauner, Maximilian F Reiser, Armin M Huber

**Affiliations:** Ludwig-Maximilian-University Munich, Campus Grosshadern, Munich Germany

**Keywords:** Left Ventricular Cavity, Gadopentetate Dimeglumine, Gadobenate Dimeglumine, Chronic Myocardial Infarction, Transmural Extent

## Introduction

Gadopentetate dimeglumine is a nonspecific gadolinium contrast agent, which distributes exclusively to the extracellular fluid space and is eliminated through the kidneys. Gadobenate dimeglumine is a second generation gadolinium agent that distributes extracellularly in the first minutes after injection, but thereafter undergoes a dual route of elimination with approximately 96% of the injected dose excreted renally and the remainder taken up by functioning hepatocytes and excreted in the bile. It has a weak reversible binding to albumin, which results in an increase of relaxation rate, compared to gadopentetate dimeglumine.

## Purpose

The two-fold higher T1 relaxivity of gadobenate dimeglumine compared with gadopentetate dimeglumine and can be used for imaging delayed enhancement in the assessment of myocardial infarction. The purpose of this study was to compare 0.1 mmol/kg gadobenate dimeglumine (Gd-BOPTA, MultiHance, Bracco Imaging SpA, Milan, Italy) with 0.2 mmol/kg gadopentetate dimeglumine (Gd-DTPA, Magnevist, Bayer-Schering Pharma AG, Berlin, Germany) in cardiac MRI.

## Methods

The study was performed in accordance to the institutional review board. Two groups of 20 patients underwent MR examinations for evaluation of chronic myocardial infarction on a 1.5 Tesla system. While group 1 received gadobenate dimeglumine at a dose of 0.1 mmol/kg, group 2 received gadopentetate dimeglumine at a dose of 0.2 mmol/kg. Single Shot IR SSFP and IR GRE sequences were used for imaging delayed enhancement (IR SSFP: TE/TR 1.1/2.2 msec, flip 50°, spatial resolution 1.3 × 1.8 × 8 mm^3^; IR GRE: TE/TR 4/11 msec; flip 25°, spatial resolution 1.3 × 1.8 × 8 mm^3^). The sizes of myocardial infarctions were measured for both contrast agents in both imaging techniques by two readers. Bland-Altman analyses were performed for each sequence and gadolinium chelate. Furthermore the transmural extent of myocardial infarction was assessed by two readers according to the 17 segment model for both contrast media and both sequences and kappa-values were calculated. Signal-to-noise ratios for infarcted myocardium, normal myocardium and the left ventricular cavity were measured and the contrast-to-noise ratios of infarcted compared to normal myocardium (CNRinf-myo) and infarcted myocardium in relation to the left ventricular cavities (CNRinf-LVC) were calculated.

## Results

The Bland-Altman plots in the assessment of infarction size did not reveal a systematic bias between the two readers. The mean difference between reader 1 and 2 was less than 0.9 cm^3^ of mean infarction volume. Assessment of interobserver agreement regarding the transmural extent of myocardial infarction resulted in kappa-values of kappa = 0.85 (IR SSFP) and kappa = 0.87 (IR GRE) in gadobenate enhanced images and kappa = 0.84 (IR SSFP) and kappa = 0.83 (IR GRE) after administration of gadopentetate. CNRinf-normal was significantly higher on the images of group 1 (gadobenate) compared to group 2 (gadopentetate) in both sequences (Single Shot IR SSFP: 18.1 ± 10.1 vs. 12.1 ± 6.7; p = 0.032 and IR GRE: 27.2 ± 5.8 vs. 19.7 ± 5.9; p = 0.005) (Table 1). The mean value of CNRinf-LVC for the group examined with gadobenate dimeglumine was lower, though not significantly, compared to the group examined with gadopentetate dimeglumien in IR GRE technique, while CNRinf-LVC for IR SSFP resulted in equal values (single Shot IR SSFP: 1.2 ± 5.2 vs. 1.1 ± 6.8; p = n.s. and IR GRE 2.4 ± 5.8 vs. 5.8 ± 7.9; p = n.s.).

**Table 1:** Table 1

Gadobenate dimeglumine		Gadopentetate dimeglumine		
Single Shot IR SSFP		Single Shot IR SSFP		p-value
TI	246.5 ± 37.2	TI	262.7 ± 25.7	0.027
SNR infarction	22.5 ± 4.3	SNR infarction	15.7 ± 8.5	0.042
SNR myocardium	4.4 ± 1.9	SNR myocardium	3.6 ± 2.1	n.s.
SNR lvc	21.3 ± 11.8	SNR lvc	14.6 ± 11.7	n.s.
CNR inf-myo	18.1 ± 10.1	CNR inf-myo	12.1 ± 6.7	0.032
CNR inf-lvc	1.2 ± 5.2	CNR inf-lvc	1.1 ± 6.8	n.s.
SD noise	2.3 ± 2.0	SD noise	2.3 ± 1.9	n.s.
**Gadobenate dimeglumine**		**Gadopentetate dimeglumine**		
**IR GRE**		**IR GRE**		**p-value**
TI	244.0 ± 29.3	TI	271.2 ± 21.6	0.019
SNR infarction	33.1 ± 9.6	SNR infarction	24.1 ± 6.1	0.001
SNR myocardium	5.9 ± 3.3	SNR myocardium	4.4 ± 1.6	n.s.
SNR lvc	30.7 ± 8.8	SNR lvc	25.1 ± 6.9	<0.001
CNR inf-myo	27.2 ± 5.8	CNR inf-myo	19.7 ± 5.9	0.005
CNR inf-lvc	2.4 ± 5.8	CNR inf-lvc	5.8 ± 7.9	n.s.
SD noise	2.5 ± 0.7	SD noise	2.3 ± 0.7	n.s.

## Conclusion

**Figure 1:**
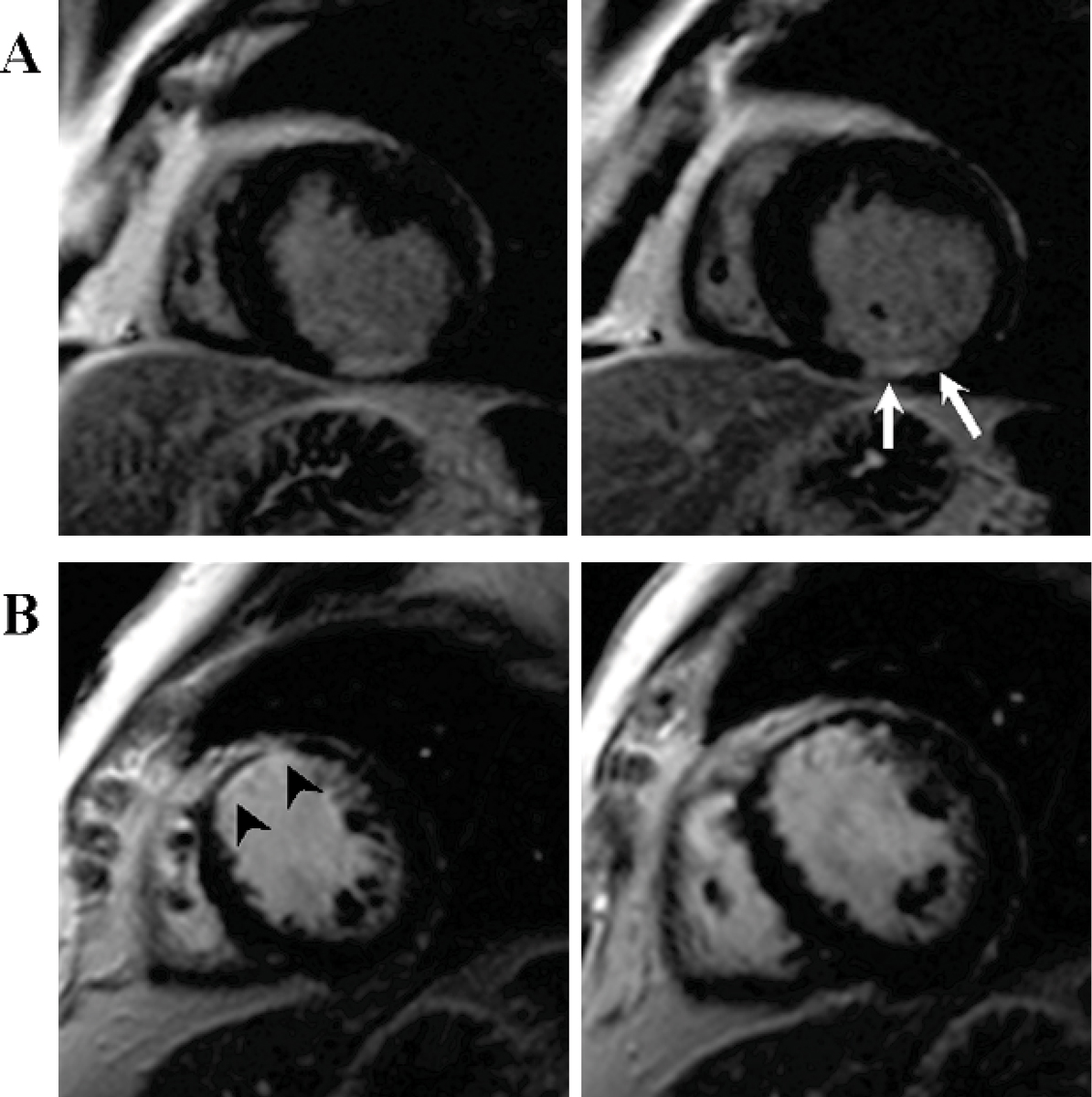
Figure 1

Low dose gadobenate dimeglumine resulted in significantly higher CNRinf-myo compared to standard dose gadopentetate dimeglumine in imaging of myocardial infarction with IR SSFP and IR GRE sequences. Demarcation of infarcted myocardium from the left ventricular cavity was equal in IR SSFP technique and not significantly different in IR GRE technique. The diagnostic value of a single dose gadobenate dimeglumine is comparable or even superior compared to a double dose gadopentetate dimeglumine for imaging myocardial infarction at least for imaging myocardial infarction in Single Shot IR SSFP technique (Figure 1).

